# Detachment of *Dunaliella tertiolecta* Microalgae from a Glass Surface by a Near-Infrared Optical Trap

**DOI:** 10.3390/s20195656

**Published:** 2020-10-02

**Authors:** Beatriz A. Juarez, Veneranda G. Garces, Beatriz Cordero-Esquivel, Gabriel C. Spalding, Kevin A. O’Donnell

**Affiliations:** 1 División de Física Aplicada, Centro de Investigación Científica y de Educación Superior de Ensenada, Carretera Ensenada-Tijuana No. 3918, Zona Playitas, Ensenada C.P. 22860, Baja California, Mexico; bajuarez@cicese.edu.mx (B.A.J.); odonnell@cicese.mx (K.A.O.); 2 lPacifica Photonics Consultants, Carretera Tijuana-Ensenada Km 99, No.1, Villa San Miguel, El Sauzal, Ensenada C.P. 22768, Baja California, Mexico; 3 División de Oceanologia, Centro de Investigación Científica y de Educación Superior de Ensenada, Carretera Ensenada-Tijuana No. 3918, Zona Playitas, Ensenada C.P. 22860, Baja California, Mexico; bcordero@cicese.mx; 4 Department of Physics, Illinois Wesleyan University, Bloomington, IL 61702-2900, USA; gspaldin@iwu.edu

**Keywords:** optical trapping, near-infrared trapping beams, *Dunaliella tertiolecta*, cell motility, detachment, photostimulus, photoacclimation

## Abstract

We report on the observation of the detachment in situ and in vivo of *Dunaliella tertiolecta* microalgae cells from a glass surface using a 1064 nm wavelength trapping laser beam. The principal bends of both flagella of *Dunaliella* were seen self-adhered to either the top or bottom coverslip surfaces of a 50 μm thick chamber. When a selected attached *Dunaliella* was placed in the trapping site, it photoresponded to the laser beam by moving its body and flagellar tips, which eventually resulted in its detachment. The dependence of the time required for detachment on the trapping power was measured. No significant difference was found in the detachment time for cells detached from the top or bottom coverslip, indicating that the induced detachment was not due solely to the optical forces applied to the cells. After detachment, the cells remained within the optical trap. *Dunaliella* detached from the bottom were seen rotating about their long axis in a counterclockwise direction, while those detached from the top did not rotate. The rotation frequency and the minimal force required to escape from the trap were also measured. The average rotation frequency was found to be independent of the trapping power, and the swimming force of a cell escaping the laser trap ranged from 4 to 10 picoNewtons. Our observations provide insight into the photostimulus produced when a near-infrared trapping beam encounters a *Dunaliella*. The microalgae frequently absorb more light than they can actually use in photosynthesis, which could cause genetic and molecular changes. Our findings may open new research directions into the study of photomovement in species of *Dunaliella* and other swimming microorganisms that could eventually help to solve technological problems currently confronting biomass production. In future work, studies of the response to excess light may uncover unrecognized mechanisms of photoprotection and photoacclimation.

## 1. Introduction

Near-infrared (NIR) optical traps have been widely used in trapping and micromanipulation of in vivo and in situ biological samples since the pioneering work of Ashkin [1]. Tightly focused laser beams can generate forces up to hundreds of picoNewtons (pN) [2] which allow trapping in vivo micro- and macro-swimmers such as microalgae [3,4,5] and sperm cells [6] for periods as long as tens of minutes without apparent photodamage. In particular, NIR beams have been used for trapping *Chlamydomonas reinhardtii* cells and measuring their flagellar swimming forces [7], propulsive forces, and flagellar beating frequencies [8]. Alternative techniques, such as the use of a micropipette for holding a single microalgae [9,10], have been used for studying flagella motility [11], changes in flagellar beat pattern [12], and adhesion [13], even though the technique is limited to relatively large flagellates with elastic cell walls [14]. Optical trapping techniques can overcome this limitation and, in principle, they could allow the observation of the complete microswimmer cell’s flagella and body motility.

*Dunaliella tertiolecta* are photosynthetic unicellular green microalgae described by Butcher [15] as an ovoid to ellipsoidal cell, l9–11 μm long and 5.5–7 μm wide, with anteriorly inserted equal flagella of 10–12 μm long. Instead of having a rigid cell wall as *Chlamydomonas*, they are surrounded by a coat which is largely glycoproteid in nature with some neuraminic acid residues as part of the molecules [16] that provides cell form flexibility. Flagella and cilia are organelles of eukaryotic cells that produce motility by repetitive episodes of bending [17] and are surrounded by a membrane that plays important roles in regulation of ciliary beat, adhesion, mechanoreception, chemoreception, thermoreception, photoreception, and cell signaling [18]. *Dunaliella* are classical models for studying the mechanisms of salt tolerance, osmotic regulation, permeability of membranes, and processes governing the biosynthesis of carotene in plants and photomovement [19,20]. They have been used recently for assessing chemical toxicity [21] and carotenoid production [22]. Current potential applications of *Dunaliella* in the field of pharmaceuticals, nutraceuticals and various industrial products have also been reported [23,24,25].

The study of photomovement and the photoregulation of movements in microorganisms is of considerable interest due to the importance of these phenomena. They are related to fundamental biological processes such as photosynthesis, photoreception, energy transformation, and membrane-mediated phenomena [26]. Understanding of photosynthesis is fundamental for microalgal biotechnology. Photosynthesis is a process of energy conversion in which light energy is trapped and converted into chemical energy [27], yet plants and algae need protection from excess light. The photosynthethic system is a dynamic flexible molecular machine that can acclimate to high irradiance changes in a matter of seconds or minutes [28]. Microalgae frequently absorb more light than they can actually use in photosynthesis. These microorganisms have evolved direct and indirect mechanisms for sensing and responding to excess light, thus surviving and photoacclimating to changes in their environment [29]. Some cells avoid absorption of excessive light by moving [20,30], as perhaps *Dunaliella* cells do, inside an optical trap, by rotating around its long axis [31]. Further, *Dunaliella* presents some advantages in their use with optical trapping technologies which include their microscopic sizes, high rate of reproduction, active motility, and photokinetic reactions.

Detachment refers to the release of microorganism cells from a solid surface into a fluid. It can be caused by external perturbations, such as fluid shear or by adding agents that remove the extracellular polymeric substance [32]. Detachment plays a significant role in biofilm formation of living microorganisms. Biofilms are important under a wide range of conditions, whether in natural environment or in infectious diseases [33]. Detachment from a glass surface of the adhered microalgae flagella is interesting because it seems to involve signals that govern the activity of the motor apparatus, flagellar activity, and the multiple functions in the regulation of flagellar beating, which can be linked to human ciliary activity studies [34].

In this work, we present observations of the detachment of adhered *Dunaliella tertiolecta* microalgae on a glass surface by a near-infrared optical trap and their subsequent behavior inside the trap. This is achieved by directing the trapping beam to a self-attached *Dunaliella* to the bottom or top coverslip of a sample chamber. We report the average detachment time as a function of trapping power and show that it is similar for *Dunaliella* detached from the bottom and top coverslips. After detaching, *Dunaliella* remained inside the optical trap. *Dunaliella* detached from the bottom coverslips were seen rotating continuously, unlike those detached from the top that did not rotate. We measured the rotation frequencies of the cells in the trap, which were found to be independent of the trapping power. Further, by decreasing the optical power to lower values, *Dunaliella* were able to move away from the trap, which permitted us to also measure their swimming forces. Our experimental results show that the 1064 nm laser wavelength can cause a photostimulus which could generate a photoresponse of the adhered *Dunaliella tertiolecta*, leading to the detachment event. The motility of the detached cells was not affected after this process. The observation of the induction of detachment in situ and in vivo using such external photostimulus could lead to understanding the genetic and molecular mechanisms that generate and regulate flagellar beating and adhesion-based signaling.

## 2. Materials and Methods

### 2.1. Cell Culture and Sample Preparation

*Dunaliella tertiolecta* were cultured in Guillard f/2 medium and reached the stationary phase under laboratory conditions at a temperature of 21 ± 1 °C, with a salinity of 34 psu (practical salinity units). The cells were kept in 125 mL Erlenmeyer glass flask with 100 mL of medium with constant white light illumination of 120 μmol m${}^{-2}$ s${}^{-1}$ (approximately 26 W m${}^{-2}$) incident intensity. The opening was covered with a cotton ball, and the flask was gently agitated daily to prevent cell sedimentation.

A volume of 14 μL, with an average of 21,000 cells, from the cell culture was placed inside a sealed shallow chamber formed by two untreated #1 coverslips separated by a 50 μm thick adhesive spacer. Both coverslips had been cleaned with methanol to eliminate residues that could affect adhesion of the flagella membrane.

### 2.2. Observations of Self-Attachment and Self-Detachment of *Dunaliella*

Immediately after sample preparation, *Dunaliella* were observed using a brightfield illumination upright microscope with a 40× objective and a 10× eyepiece. *Dunaliella* were seen swimming horizontally at an average velocity of approximately 120 μm s${}^{-1}$ similar to that reported for Dunaliella bioculata [26], with some immobilized on the top or bottom coverslips of the shallow chamber. The average velocity was obtained by tracking a total of 20 cells across the field of view (FoV), and taking the average of the cell displacement divided by the tracking time. In general, *Dunaliella* in the initial and exponential growth phases were not adhered to the glass surfaces, contrarily to *Dunaliella* in the stationary growth phase that appeared to be adhered strongly without being able to detach for periods of time as long as 30 min. Keeping the initial FoV, the self-attached cells were counted every 15 min over a period of 3 h. Significant ultrastructural differences are seen between the stationary and exponential growth phases of *Dunaliella tertiolecta*. Notably, there is an increase in lipids in the stationary growth phase cells [35].

Even though the flagella are thin (0.2 μm in diameter) [20], it was possible to see the principal bends of both flagella [36,37,38] near the basal bodies, adhered to the coverslips, by using a 60× objective. We could also observe the flagella tips moving up and down, which was accompanied in some cases by a pivoted swaying of the *Dunaliella* body.

### 2.3. Optical Trapping System

The optical trapping system was based on a Nikon Eclipse TE inverted microscope with white light illumination. The 1064 nm wavelength trapping laser (Cobolt, 2 W power, CW, linearly polarized) was sent into the back aperture of a 60 × 1.49 NA TIRF objective and focused on the sample. Previous works have also used high NA objectives for trapping green biflagellated *Chlamydomonas* microalgae [3,7,8]. This laser wavelength was used in all our experiments shown here because it has proven to be ideal for trapping living cells of the yellow-green microalgae *Trachydiscus minutus* without producing visible photodamage [39]. In order to optimize the far-field optical trapping power, the back aperture of the objective was underfilled with a filling ratio of 0.79 [40]. To control the incident power sent to the cells, a half-wave plate and a polarizing beamsplitter were used. The system was also equipped with a piezoelectric microscope stage that allows the calibration of the optical trapping forces using the drag force method [41]. A CCD camera (Pulnix TM-7EX) of 30 frames per second (fps) was located at the eyepiece port of the microscope, thus providing a view from the bottom of the sample chamber for video recording all experiments reported here.

### 2.4. Induced Detachment of *Dunaliella*

A sample chamber was placed on the stage of the optical trapping system after 30 min of its preparation, and it was seen that a significant number of *Dunaliella* were attached either to the bottom or top coverslip. The experiments were done at room temperature and with constant white light illumination from the top. An optical trapping power range from 55 mW to 200 mW was used for achieving the detachment. Before initiating the detachment, the trapping laser beam was positioned near the center of the field of view. By observing the reflection of the beam waist on the bottom glass surface, the TIRF objective was moved up 12 μm (when detaching from the bottom); similarly, by observing the reflection of the beam waist on the top glass surface, the TIRF objective was moved down 20 μm (when detaching from the top). These two positions of the objective were selected for being able to keep the cells inside the trap and observing their behavior after detachment. Recently, Català et al. [42] have reported the influence of experimental parameters on the laser heating of an optical trap. They had estimated that for a typical laser power of about 200 mW, assuming that the heating of the cells is similar to that of water, the maximum local temperature could rise by approximately 8 ${}^{\circ }$C. Increasing the distance of the trapping position from the bottom coverslip of the sample chamber causes a strong decrease in the optical forces [41]. In our experiments, the optical forces exerted by the laser to the attached cells were five times weaker for cells attached on the top than for those attached on the bottom [31]. With the beam blocked, the microscope stage was moved horizontally until a *Dunaliella* was found and positioned in the optical path. In the experiments reported here, we have chosen only cells that had the principal bends of their flagella adhered to the coverslip as in a gliding position [43]. This position allows them to move their flagellar tips and their body as will be seen in the videos discussed later in Section 3.2. Then, the trapping laser was unblocked. We recorded the full detachment event with the CCD set at 30 fps. The time from when the laser beam was unblocked until the detachment was observed was defined as the detachment time τ. This information was extracted from the video recorded.

### 2.5. Rotation and Swimming Forces of *Dunaliella* after Being Detached

After being detached, the *Dunaliella* remained motile inside the optical trap, and their behaviour was also recorded. Cells detached from the bottom coverslip were seen rotating and moving up away from the glass surface until they reached an equilibrium position inside the optical trap. Their rotation was in a counterclockwise direction along their own axis. This appears to be the same swimming rotation of free swimming *Dunaliella*, but the cells are inside the trap and thus remain rotating at the same position. On the other hand, the cells detached from the top coverslip did not rotate because they were pushed against the coverslip by the trapping beam. Thus they were kept inside the trap with their long axis perpendicular to the optical axis. For the cells detached from the bottom coverslip, the average rotation frequency as a function of the laser trapping power was obtained by recording the rotation of five cells for each trapping power. The cells rotation frequencies were obtained from the recorded video, as in previous works [8,31].

*Dunaliella* were always seen rotating inside the optical trap as long as there was enough optical power for keeping them trapped. By reducing the trapping power, the trapped *Dunaliella* were able to escape from the trap. The values of the optical power were then recorded and, by interpolating the trapping power-force calibration curve of our optical trapping system obtained previously [31], the swimming forces were estimated.

## 3. Results and Discussion

### 3.1. Monitoring the Amount of *Dunaliella* Self-Attached on Glass Coverslips

Surface interactions of motile cells play crucial roles in a wide range of microbiological phenomena such as the formation of biofilms [33] and during the fertilization of mammalian ova [44]. The microswimmers, such as flagellated microalgae, move within a structured environment often confined by solid surfaces [45]. Adhesion to surfaces via the microalgae’s flagellar membrane is a natural process which they do to fulfill reproductive functions or as an alternative motility mode by surface gliding [46]. Even though the *Dunaliella* were seen with their flagella’s principal bends adhered to the coverslip and oriented at 180${}^{\circ }$ to one another, a typical characteristic of gliding, we did not observe this phenomenon here.

**Figure 1:**
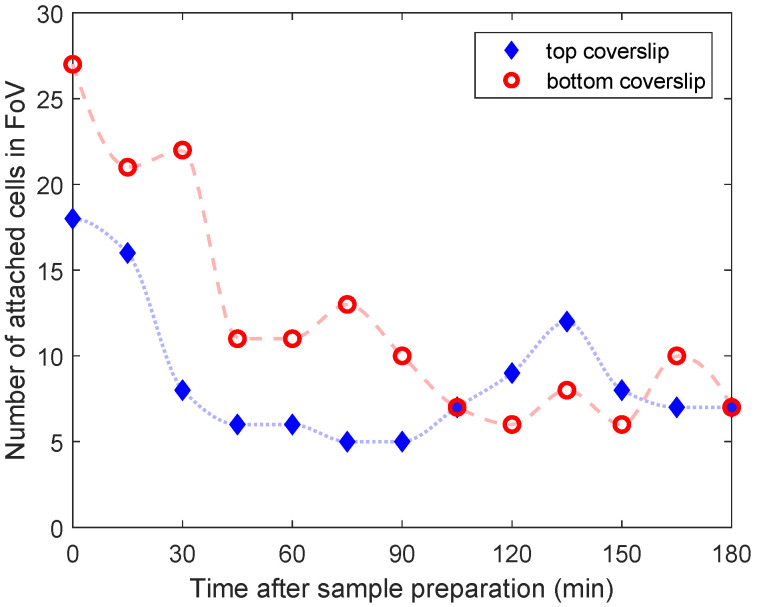
Number of attached cells in the FoV as a function of the time since sample preparation. The filled diamonds indicate the cell counts on the top coverslip, while the empty circles are for those on the bottom coverslip.

The number of *Dunaliella* self-adhered on an area of the glass surfaces (bottom and top coverslips) of a shallow chamber was monitored as a function of time passed after sample preparation. The results are shown in Figure 1. Here, it is notable that the maximum number of attached cells, on both top and bottom coverslips, occurred just after the sample preparation. This occurs presumably because at the end of the sample preparation, the *Dunaliella* cells are physically pushed towards the surfaces in order to seal the sample chamber. It is also remarkable that the number of adhered cells always kept changing without the influence of external photostimulus or optical forces. In the 180 min of monitoring, some cells can be self-detached (decreasing the number of adhered cells), and other cells can be self-adhered (increasing the number of adhered cells). In general, in our shallow sample chamber, *Dunaliella* could always be found attached to the glass during the time period for which the experiments reported here were run.

To our knowledge, studies of *Dunaliella* self-attachment and self-detachment on solid surfaces have not been addressed in previous works. Instead, characterization of surface colonization of *Dunaliella* on diverse materials [32] and the observations of *Dunaliella* behavior near surfaces of a plastic cuvette [47] have been studied. In this last work, *Dunaliella* were seen self-attached to the vertical walls of the cuvette, being more densely packed at the top near the liquid-air interface. In general, it is believed that the bacterial adhesion could occur in an initial approach followed by physical bonding to the surface [48]. During attachment, the membrane of the *Dunaliella’s* flagella, which is a heterogeneous complex molecular structure, is in contact with the glass surface. The cell attachment is a complicated process and is related not only to the surface energy, but also the physical, electrical, chemical, and biological characteristics of the microorganisms and their interrelationship with the solid substrate. We believe that the cells have detached themselves due perhaps to optimize the photosynthetic efficiency in conjunction with survival.

Development of biofilms occurs often on surfaces exposed to an aqueous environment. The self-adhesion of *Dunaliella* on glass surfaces may lead to biofilm formation [33], which is relevant in the design of photobioreactors [49]. Thus the study of self-adhesion and self-detachment on glass surfaces of microalgae could be important for biomass production and could also help to understand the flagella function in sensing and response in the development of biofilms.

### 3.2. Induced Detachment of *Dunaliella* by a NIR Trapping Beam

**Figure 2:**
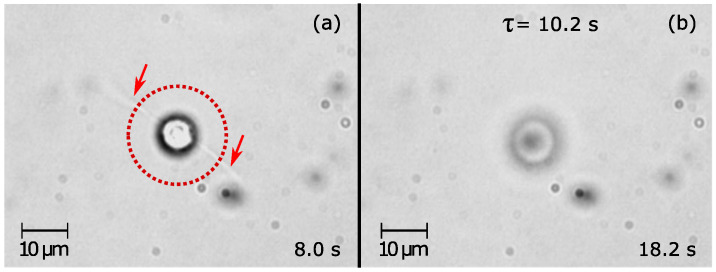
Two frames taken from Video S1 showing the initial and final positions of the *Dunaliella* in a detachment event induced by the trapping beam. (**a**) The adhered *Dunaliella* in the red dashed circle aligned to the trapping beam after unblocking the beam. The adhered flagella are indicated with the red arrows. (**b**) At 18.2 s (τ = 10.2 s), the cell was detached and moved upwards, appearing here out of focus but still remaining in the optical trap.

To induce the detachment of *Dunaliella* using a 1064 nm wavelength trapping beam, a *Dunaliella* attached to a glass coverslip surface was selected and positioned at the location of the blocked trapping beam. Later, the trapping laser was unblocked and the time that the detachment occurred was recorded. We have recorded detachment events for *Dunaliella* adhered to the bottom (Supplementary Materials Video S1) and top (Supplementary Materials Video S2) urfaces. Figure 2 shows two frames taken from Video S1. Figure 2a shows a *Dunaliella* just after unblocking the trapping beam set at 200 mW. Here, the shadow of the flagella can be seen as thin lines protruding from the body (indicated by the arrows). In Video S1, it is possible to see how after the beam is unblocked (8.0 s timestamp), the cell aligns its body to the optical beam axis and seems to slightly increase its flagellar tips motion until detachment occurs (18.2 s timestamp), which is noted by the cell moving up away from the coverslip and thus going out of focus (Figure 2b). Thus, the detachment time was τ = 10.2 s. The behavior of the *Dunaliella* after being detached until its release is also shown in Video S1. As can be seen, after detachment the cell kept rotating in a counterclockwise direction inside the optical trap even when the trapping laser power was decreasing from 200 mW to 94 mW (28.0 s to 37.9 s timestamp). This is an indication that *Dunaliella* always remain rotating in the same direction for the range of optical powers used here.

The rotation of the green microalgae *Chlamydomonas* in an optical trap has been reported in previous works [3,7,8,50,51]. In 2009, Foster [30] reported that *Chlamydomonas* always rotate in a counterclockwise direction because of the twofold rotation symmetry of the cilia and the tilt of their power stroke. At the end of Video S1, when the optical power decreased to 73 mW (38.0 s timestamp), it is possible to see the cell swimming away from the optical trap (38.9 s timestamp). This escape of the *Dunaliella* indicates that the cells did not lose their ability to swim after being detached by the trapping laser.

A detachment event of a *Dunaliella* attached on the top ceverslip is shown in Video S2. It is possible to see how the cell starts shaking its body and its flagellar tips after unblocking the trapping beam until its detached (5.9 s to 16.6 s timestamp). Here, after being detached, the cell is kept trapped with its body perpendicular to the optical axis but without rotating along its long axis (16.7 s to 36.2 s timestamp). Finally, the cell escaped from the optical trap at 135 mW optical power (36.3 s timestamp), showing once again that it could swim after being detached by the trapping beam.

**Figure 3:**
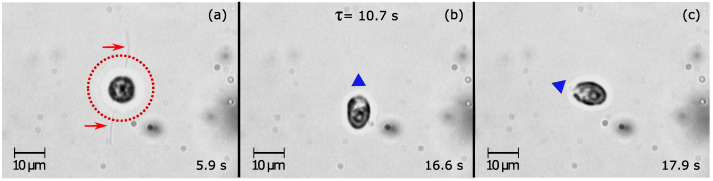
Three frames taken from Video S2 showing induced detachment of the adhered *Dunaliella* from the top coverslip. (**a**) The adhered *Dunaliella* highlighted in the red circle is positioned in the path of the laser beam, and at t = 0 s the beam is unblocked. Its adhered flagella are indicated with the red arrows. (**b**) At the moment of the detachment (τ = 10.7 s), and (**c**) 1.3 s later, the cell shows a different orientation in the optical trap as indicated by the by the blue triangle mark.

Figure 3 shows three frames taken from Video S2. A *Dunaliella* attached to the top coverslip with its long axis parallel to the beam axis and its flagella is shown in Figure 3a. At this moment the trapping beam is unblocked. Figure 3b shows when the attached *Dunaliella* physically separates its flagella from the coverslip (τ = 10.7 s) and moves 90° being now with its long axis perpendicular to the optical axis. Then, 1.3 s later, the cell was moved in a different position (see Figure 3c) showing its detachment from the coverslip. In order to understand why the cells detached from the top were not moving down to the point of stable equilibrium located close to the beam waist [52], we have trapped dead and/or deflagellated cells and place them in the optical path. Interestingly, these cells were always guided and caught near the beam waist immediately after unblocking the trapping beam. The fact that after detachment the cells were not caught near the equilibrium position indicates that the cells avoid to be guided towards the laser beam.

The event of detachment was achieved for every single attached *Dunaliella* exposed to the trapping laser. In general, we observed *Dunaliella* photoresponses to the incident trapping laser which could cause a photostimulus, leading to flagellar tips and body motions, and eventually to a detachment event. Fast flagella motion has been observed in biflagellated *Isochrysis* sp. microalgae, after being optically trapped with a 785 nm wavelength laser beam, leading to a deflagellation event [5]. There, the authors also reported that three different types of flagellated microalgae: *Isochrysis* sp., *Dunaliella salina*, and *Haematococcus pluvialis* were optically trapped with a 1064 nm CW laser beam without deflagellation. Thus, it seems that the photostimulus induced by a trapping laser could depend on the cell type and laser wavelength.

### 3.3. Induced Detachment Time Measurements

**Figure 4:**
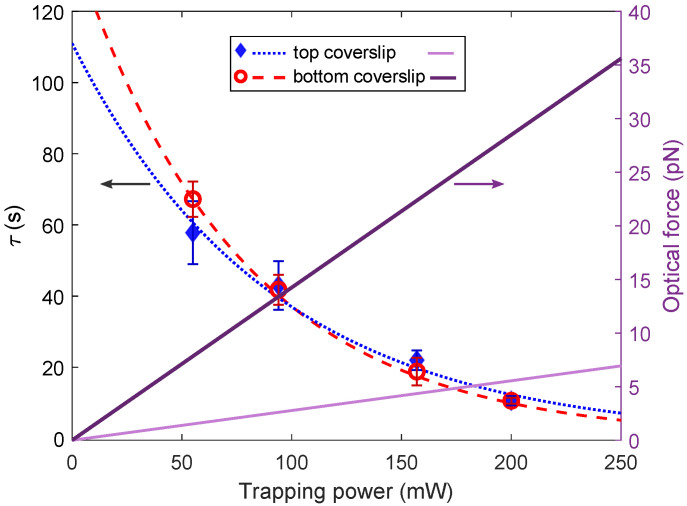
Detachment time τ (left vertical axis) and optical forces (right vertical axis) of the *Dunaliella* adhered to top or bottom coverslips as a function of the trapping power. The filled diamonds correspond to the detachment time from the top coverslip, and the open circles to the detachment time from the bottom coverslip. The dashed lines are the least squares fits to decaying exponential obtained using the Levenberg-Marquardt algorithm. The solid lines (dark and light) are the optical forces exerted to the cells attached to the bottom and top coverslips, respectively.

The detachment time τ obtained for *Dunaliella* adhered to either bottom or top coverslips as a function of the trapping power is shown in Figure 4. We were able to detach *Dunaliella* using trapping powers lower than 55 mW and higher than 200 mW but the cells were not always trapped after detaching. Thus data outside the power range from 55 mW to 200 mW were not included here. Each value corresponds to an average of five measurements of different *Dunaliella*. The error bars were calculated by taking the standard deviation of a set of data for a given power and dividing by the square root of the number of cells. It is notable that the error bars are much larger for small trapping powers. As we can see, τ decreases for higher optical power, being near 60 s for 55 mW, while reducing to 12 s for 200 mW. The mechanisms of sensing and signaling the excess light by *Dunaliella* cells seem to vary depending on the amount of optical power received. The mechanisms of sensing and signaling the excess light by *Dunaliella* cells seem to vary depending on the amount of optical power received and therefore on the possible heating induced in the cells [42]. Using the Levenberg-Marquardt algorithm, a decaying exponential function to each data was fitted (dashed lines with a same fit value of $R^{2}=0.98$) as $\tau_{top}$ *(P)* = 111 *exp* (−0.011*P*) and $\tau_{bottom}$ *(P)* = 139 *exp* (−0.013*P*). These functions indicate that even without having the trapping laser power on, a *Dunaliella* adhered to the top and a *Dunaliella* adhered to the bottom can be detached after 111 s and 139 s, respectively. This result is in agrement with our previous results shown on Figure 1 where the attached cells were able to self-detach without any trapping laser. The values of −0.011 and −0.013, multiplying *P*, could be weight factors that depend on the *Dunaliella* capability to absorb, photosense and photoreact to the laser trapping power. The two fitted functions to the data have small discrepancies that fall within the calculated errors in the data sets, so thus we could say that they are similar in the trapping power range of our study, and then indicate that the detachment time τ is independent of the position of the attached cell.

Figure 4 also shows the optical forces (right vertical axis) exerted on the attached cells by the laser as a function of the trapping power (light and dark solid lines for top and bottom coverslips, respectively). Here it is possible to see what was mentioned in Section 2.4 that, in our system, the optical forces are up to five times weaker when the detachment is induced to the attached cells on the top coverslip [31]. Nevertheless, the detachment time seems to be similar regardless of the position of the attached cells, in particular at the highest optical power of 200 mW. The optical forces are directly proportional to the trapping power, thus it is also possible to conclude that the trapping forces have no significant role in the exponential decay of the detachment time. Perhaps as both attached cells received the same amount of optical power, the NIR laser causes to them similar photochemical and photothermal effects. The phenomenon of detachment reported here was without doubt caused by sending the trapping laser beam to the attached *Dunaliella*, which immediately moves its body out of the contact points and its flagellar tips up and down. *Dunaliella* cells might have been evolved to photoprotect itself balancing the amount of absorbed NIR laser beams and its utilization using many of its molecules and enzymes through photosynthesis [53].

In order to determine the mechanism that could produce the detachment event, the trapping laser transmittance *(T)* of 20 *Dunaliella* attached to the bottom coverslip were measured using a photodiode placed in a conjugate plane of the condenser back aperture [54]. A value of *T* = 0.95 ± 0.01 was found indicating that absorption and scattering is produced by the cells. Our *Dunaliella* cells, in the stationary phase, accumulate a significant amount of β -carotene [55] which could contribute to the absorption of the trapping laser. The detachment of *Dunaliella* cells have been induced with different optical powers, even if 200 mW of power would induce nearly four times more heating than a power of 55 mW [42,56], thus we believe that the detachment event could not be due to the increment of temperature *per se*. Perhaps different changes in temperature of the cell could generate distinct signaling pathway branches that triggers the detachment.

Why does *Dunaliella* moves its flagellar tips faster when it is illuminated by the 1064 nm trapping laser beam? This is an interesting question. In the past, an attempt to establish a cause and effect relation between visible light stimulus and response in microorganisms was addressed in a number of investigations [57,58]. For instance, Buder in 1917 [57] proposed the probable location of the photosensitive system in *Euglena*, and described the relationship between flagellar beating and the position of stigma relative to the source of light. An excellent historical overview of research and current state of the art of photomovement of algae can be found elsewhere [20]. Change from ciliary (asymmetric breaststroke) to flagellar (symmetric whip-like) beating mode in response to green light stimulus in *Chlamydomonas* has been correlated with intraflagellar calcium generated causing flagellar motility [59,60]. Recently, Kreis et al. [13] showed that the adhesion of *Chlamydomonas* to the surfaces via their flagella is switchable with light. The induction of detachment from a glass surface of *Dunaliella tertiolecta* using a 1064 nm laser beam has not been reported before, and neither have any photoresponses to photostimulus causing fast *Dunaliella’s* flagellar tips motion using NIR laser beams. It is possible that our cells have efficient biochemical defense mechanisms to counteract the absorbed excess of light caused by our NIR optical trap. The mechanisms that seem to cause the detachment events could be a combination of photochemical and photothermal effects. We speculate that without being able to move, the cells detach in a short time (few to tens of seconds) due to survival and photoacclimation [53], and during relatively long time (tens of minutes or days), the cells could eventually acclimate to excess light [24].

### 3.4. Rotation within the Optical Trap and Swimming Forces of Detached *Dunaliella*

The rotation of a single detached *Dunaliella* and its subsequent levitation in an optical trap has been observed previously [31]. In the current report, the rotation frequencies of randomly selected detached *Dunaliella* were measured to see if these cells showed changes in their rotation frequency that could be attributed to photodamage during the induced detachment event. Optically trapping of living cells can cause photodamage which depends on the cell type, laser-trapping wavelength, and optical power. Previous studies of *Dunaliella tertiolecta* using a 1064 nm optical trap were reported without noticing any apparent photodamage for an optical power range of 40 mW to 320 mW [31].

**Figure 5:**
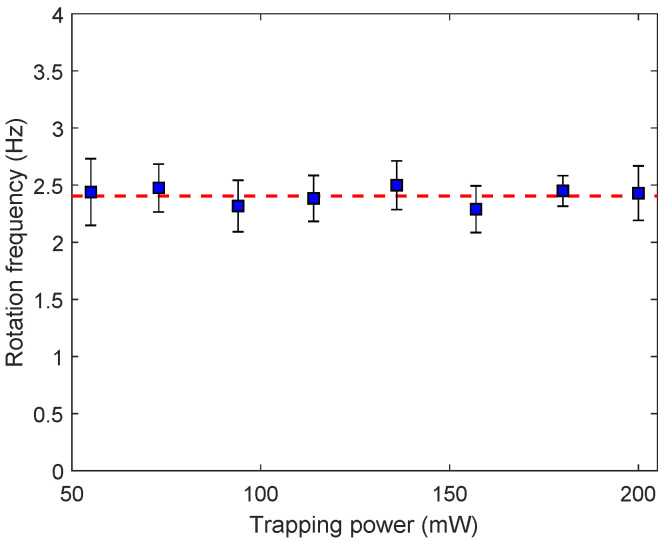
Rotation frequency of *Dunaliella* as a function of the trapping power. The dashed line is the average rotation frequency of the detached *Dunaliella* for the whole range of optical powers.

The rotation frequency of five different detached *Dunaliella* were measured as was described in Section 2.5. Figure 5 shows the average rotation frequency as a function of the trapping power. The error bars were calculated as was described in the Section 3.3. The average rotation frequency of the detached *Dunaliella* was of 2.4 Hz ± 0.3 Hz for the whole range of optical powers from 55 mW to 200 mW. This result implies that the trapping laser did not cause any apparent change in the rotation frequency of the *Dunaliella* after being detached. Also it seems that the optical forces were not driving the trapped cell to rotate, as was observed in the case of *Chlamydomonas* cells [3]. Instead, it seems that *Dunaliella* cells have evolved mechanisms for sensing and responding to the excess trapping light, perhaps by avoiding absorption using its photosynthetic system which is a dynamic flexible molecular machine that can acclimate to high irradiance changes in a matter of seconds or minutes [28].

Later, with the detached *Dunaliella* rotating inside the optical trap, the swimming forces were estimated as described in Section 2.5. A few randomly selected detached *Dunaliella* were again chosen for performing the swimming force measurements. Typical results are also shown at the end of the Videos S1 and S2. In Video S1, the rotating *Dunaliella* escaped from the trap when the optical power was set at 73 mW (38.9 s in Video S1), which corresponds to a maximum lateral force exerted by our trapping system of 10 pN (see [31]). Video S2 shows when a *Dunaliella*, trapped near the top surface of the sample chamber, moved away from the trap when the laser power was set at 136 mW (36.3 s in Video S2). At this power, the maximum lateral force exerted by the trapping system was estimated to be 4 pN. Freely swimming cells were also trapped and their swimming forces were calculated. Using five randomly selected cells, an average swimming forces of 1.7 pN was estimated.

It is thus seen that after detaching, the cells rotated at the same frequency independent of the trapping power. The cells also preserved their motility upon release, showing similar swimming forces of detached and free swimming cells. Thus we can affirm that, in the range of optical powers reported here, there was no apparent photodamage observed in the motility after inducing detachment. In fact, we can speculate that after detachment, even with the highest power of light of 200 mW which could induce an increase of temperature of 8 ${}^{\circ }$C [42], *Dunaliella* cells had similar behavior inside the optical trap and thus showed photoacclimation to trapping powers in the range of 55 mW to 200 mW.

## 4. Conclusions

We have demonstrated the detachment of *Dunaliella tertiolecta* from glass coverslip surfaces by using a 1064 nm wavelength trapping laser beam. *Dunaliella* in the stationary growth phase were placed in a shallow sample chamber, and were seen self-attached on either bottom or top glass surfaces. We have observed and recorded the induced detachment event in situ and in vivo. This event was caused by sending the trapping laser beam to the attached *Dunaliella* which immediately had a photoresponse or photoreaction, by changing its flagellar tips and body to slightly faster movements, which eventually led to its detachment. The detachment time is faster for higher trapping powers. By obtaining similar induced detachment times for *Dunaliella* attached on bottom or top coverslip surfaces, we suspect that the detachment appears to be induced by the absorption of the laser through the cell, instead of due solely to the optical forces exerted on the cell. In general, the light absorption, the rate of electron transport and the carbon metabolism are all synchronized to maximize the yield of photosynthesis in *Dunaliella* cells [24], but when the absorption of light in excess of that required for the saturation of photosynthesis, it can inevitably generate highly reactive oxygen species as byproducts that can cause oxidative damage to the photosynthetic apparatus [61]. The cells used in our experiments contain high levels of β-carotene which could play a protective role as a singlet oxygen scavenger [62]. Because our trapping laser seems to perturb the photosynthesis system in the attached *Dunaliella* cells, their photoresponses to this excess light, directly by photoreceptors or indirectly through biochemical and metabolic signals, could cause the detachment event. All living photosynthetic microorganisms integrate signals from multiple sensors in order to survive and acclimate to any environmental change such as light intensity, temperature, nutrients, and physical walls. In nature, the modulation of any one of these external conditions rarely occurs independently of the others [63].

Because the detachment of the cells involve changes in the flagella motility, we believe that measurements like those reported here could lead to a better understanding of the photoreception of biflagellated microalgae to NIR laser beams. It could be also possible to find the location of the photoreceptor in *Dunaliella*. In fact, it is known that changes in the concentration of Ca${}^{2+}$ ions near the flagella modulate their beating [20]. The photoreception of NIR light could be viewed as an excitation of certain proteins due to the absorption of the light by some photoreceptor molecules, and perhaps the conformational alterations in the protein could lead to the activation of ion channels. In the future, the integration of multiple signal in coordination with the excess NIR laser power may uncover unrecognized mechanisms of photoprotection.

In addition, we reported how the trapping laser did not cause any apparent change in the rotation frequency of the *Dunaliella* after being detached, showing nearly constant counterclockwise rotation frequency for the range of trapping powers used here. We also showed that, by decreasing the trapping power, the cells were released and moved away from the trapping site. Notably, every detached *Dunaliella* was able to escape after being trapped for periods of time ranging from 50 s to 120 s. We also measured the swimming forces, which ranged between 4 pN and 10 pN. These results suggest that, after detachment, the *Dunaliella* cells were rotating and swimming without showing photodamage. Therefore, we believe that there was no apparent photodamage observed in the motility of the detached cells; instead it seems that *Dunaliella* cells could undergo molecular physiological changes in order to acclimate to the NIR trapping beam.

Detachment induced by a 1064 nm wavelength trapping laser could involve cellular and molecular responses. Observation in situ of individual *Dunaliella* detaching from a glass surface using a trapping laser undoubtedly has significant advantages. For instance, it could be easily combined with fluorescent imaging techniques to study molecular intraflagellar transport [64,65]. Ultimately, it may contribute to understanding the rhodopsin-mediated sensory transduction events in green flagellated algae for phototaxis and photophobic response. Intraflagellar transport, flagellum-based motility, flagella outgrowth, flagellar beating patterns, and the induction of detachment (as demonstrated here) are complex processes which could be connected to a common photostimulus. We believe that our experimental results provide new knowledge towards a better understanding of behavioral photoreaction of green flagellated microalgae induced by NIR trapping beams.

## Supplementary Materials

The following are available online at https://www.mdpi.com/1424-8220/20/19/5656/s1, Video S1: Detachment event induced by the trapping beam to an adhered cell on the bottom glass coverslip of a sample chamber, Video S2: Detachment event induced by the trapping beam to an attached cell on the top glass coverslip of a sample chamber.

## Abbreviations

The following abbreviations are used in this manuscript:

NIR	Near infrared
PSU	Practical salinity units
MINS	Minutes
CCD	Charged coupled device
FoV	Field of view
FPS	Frames per second
