# Supplementary material for: Detachment of Dunaliella tertiolecta Microalgae from a Glass Surface by a Near-Infrared Optical Trap
Source: Sensors (Basel). 2020 Oct 2;20(19):5656. doi: 10.3390/s20195656 (PMC7582954; doi:10.3390/s20195656)
Supplement: Supplementary file 1 [file sensors-20-05656-s001.zip › Supplementary Videos Captions.pdf]

Captions of Supplementary material (Video 1 and Video 2)

Video 1. Detachment event induced by the trapping beam to an adhered cell on the bottom glass coverslip of a sample chamber.

Video 2. Detachment event induced by the trapping beam to an attached cell on the top glass coverslip of a sample chamber.
